# Ultrasound-versus visual-guided transversus abdominis plane block prior to transabdominal preperitoneal ingunial hernia repair. A retrospective cohort study

**DOI:** 10.1016/j.amsu.2020.09.017

**Published:** 2020-09-22

**Authors:** C. Paasch, J. Fiebelkorn, G. De Santo, N. Aljedani, P. Ortiz, U. Gauger, K. Boettge, S.H. Full, S. Anders, M. Hünerbein

**Affiliations:** a Department of General, Visceral and Cancer Surgery, Helios Klinikum Berlin-Buch, Schwanebecker Chaussee 50, 13125, Berlin, Germany; b Humboldt University, Berlin, Germany; c No Insurance Surgery, 9121 W Russell Rd Ste 115, 89148, Las Vegas, United States; d Department of Anesthesiology, Helios Klinikum Berlin-Buch, Berlin, Germany

**Keywords:** Transversus abdominis plane, TAPP, Inguinal hernia repair, Ultrasound-guided TAP block, Visual-guided TAP block

## Abstract

**Background:**

The transversus abdominis plane block is a regional anesthesia technique. Recently, its impact on early chronic pain and the cumulative need of analgesic medication following inguinal hernia repair is being monitored. In terms of effectiveness and patient safety, it remains unclear whether the approach should be conducted preoperatively through ultrasound guidance, or through intraoperative visual guidance.The study at hand aims to provide more evidence on this topic.

**Methods:**

A monocentric retrospective matched pair analysis was performed. The intraoperative visual guided and ultrasound guided -transversus abdominis plane block prior to inguinal hernia repair in transabdominal preperitoneal technique were consecutively compared in regard to analgesic effectiveness and complication rate. The data of individuals who were operated on from June 2007 to February 2019 were analyzed. The matching criteria were ASA-Score, Gender, Age ( ±6 years), and hernia size (<1,5 cm, 1,5-3 cm, >1,5 cm).

**Results:**

A total of 116 patients were enrolled. Both groups were homogenous in terms of age, gender contribution, body mass index, ASA-Score, hernia type, and size. The pain score at the postoperative anesthesia care unit was lower in the ultrasound-guided-transversus abdominis plane group without being statistically significant (VAS-Score: 0.67 vs.0.84). Patients of the ultrasound-guided-transversus abdominis plane group received significantly less metamizole on the day of operation (1.29 g (0.96) vs. 1.68 g (0.70), p = 0.015).

**Conclusion:**

Due to our findings, we assume that the ultrasound-guided-transversus abdominis plane -Block may reduce postoperative pain and analgesic consumption more effectively than the visual-guided-transversus abdominis plane lock. Further prospective clinical trials are mandatory.

## Highlights

• The TAP-Block can be performed ultrasound- and visual-guided.
• No TAP-Block related complications appeared among our 116 individuals.
• Ptients who received the ultrasound-guided transversus abdominis plane block needed significantly less metamizole on the day of operation.

## 1 Introduction

Early postoperative pain following laparoscopic inguinal hernia repair (IHR) occurs frequently [1]. It impacts early ambulation, physical recovery, and, as a known risk factor, the rate of chronic pain [[1], [2], [3]]. Hence, local anesthetic approaches methods have been reviewed to improve postoperative pain level.

Several authors demonstrated that subfascial/subcutaneous local infiltration and anesthetic field blocks reduce early postoperative pain, and the need of analgesic medication following open IHR [4,5]. Moreover, a meta-analysis revealed proficient analgesic effects of the ilioinguinal/iliohypogastric nerve block postoperatively [6].

Regarding laparoscopic IHR (transabdominal preperitoneal (TAPP) and total extraperitoneal (TEP) technique), a total of 6 studies involving 1089 patients demonstrated a statistically compelling transversus abdominis plane (TAP) block impact on pain reduction and painkiller consumption [2,[7], [8], [9], [10], [11]]. This practice was first described in 2001 by Rafi et al. [12]. In this intervention, a local anesthetic amide type agent (rupivacaine, bupivacaine) is injected between the rectus abdominis muscle and the posterior rectus sheath. The sensory nerves innervating the abdominal wall originating from T7 to L1 (intercostal, ilioinguinal, subcostal, and iliohypogastric nerves) are the target of the TAP block [12].

In terms of effectiveness and patient safety, it remains unclear whether the method should be conducted preoperatively through ultrasound-guidance (UG), or intraoperatively through visual-guidance (IVG). Until now no study has been conducted to compare both approaches to each other. We assume that the IVG-TAP block may be more effective. Ultrasound imaging may facilitate a more precise injection.

Thus, the study at hand aims to provide more evidence on this topic.

## 2 Patients and methods

A monocentric retrospective matched pair analysis was performed. The UG-TAP block prior to primary IHR in TAPP technique was compared to the IVG-TAP block in terms of analgesic effectiveness, use, and complication rate. The data of individuals who were consecutively operated on from June 2007 to February 2019, were analyzed. The matching criteria were ASA-Score, Gender, Age ( ±6 years), and hernia size (<1,5 cm, 1,5-3 cm, >1,5 cm).

The data were collected at the xxx hospital xxx (xxx) between April and May 2020. The study was approved by the Ethics Committee of the ‘Ärztekammer xxx’ (Medical Association xxx) in September 2019 (Eth-12/19), and conducted in accordance with the ethical standards of the Helsinki Declaration 1975.

The study was registered with the German clinical trial registry DRKS (DRKS00021679). No funding has been received. The work has been reported in line with the STROCSS criteria [13].

The study is based on the patients’ data available from their files. The time of their hospital stay has been analyzed. We did not perform a systematic follow-up.

The study has been reported in line with the STROCSS criteria [13].

### 2.1 Inclusion criteria

Patients who received an UG-and IVG-TAP block prior to primary laparoscopic IHR in TAPP technique were included. Being over the age of 16 and physical status of I-III based on the American Society of Anesthesiologists criteria were further inclusion criterias.

### 2.2 Exclusion criteria

Patients who underwent a conversion to open surgery, being under the age of 16, allergy to local anesthetics and a recurrent hernia were exclusion criterias.

### 2.3 TAP block technique

The TAP block was performed through preoperative ultrasound and intraoperative visual guidance. In 2015, the VG approach was chosen, because the UG-TAP block appeared to be more time consuming. Before 2015, all patients received an UG-TAP block.

#### 2.3.1 Intraoperative visual-guided TAP block

Under direct visualization using the laparoscope at the beginning of the operation, the TAP block was conducted. While the abdominal cavity was insufflated, the surgeon palpated the lateral border of the rectal sheath, ensuring adequate lateral placement. Next, a 19 G needle was inserted percutaneously above the iliac spine, inferior to the costal margin at the lateral end of the surgical field. The needle was advanced through the internal and external obliques. Laparoscopically, we ensured that the peritoneum was nearly penetrated. We then tried to retract the needle for 2 mm to reach the proper layer. A small amount of local anesthetic was injected between the internal oblique and transversus abdominis. Appropriate dispersal throughout the layer was visually affirmed prior to the injection of the entire extent of local anesthetic. The same procedure was conducted on the contralateral side in the event of a bilateral inguinal hernia. The amount of local anesthetic for unilateral tap block consisted of 75 mg ropivacaine mixed with 20 cc of normal saline. Twice the amount was used in the prospect of a bilateral inguinal hernia. The process took approximately 1 min. A systematic time recording was not carried out.

This technique took approximately 1 min (each side). A systematic time recording was not performed.

#### 2.3.2 Ultrasound-guided TAP block

After covering and disinfecting the appropriate integumentary field, the UG-TAP block was conducted under anesthesia through ultrasound guidance (body habitus dictated linear high-frequency 12 MHz or curvilinear abdominal 6 MHz probe use) using a 22G needle, and a standard lateral approach was performed preceding the operation.

The medial head of the ultrasound probe was positioned on the anterior wall at the level of the umbilicus, with adjustments made to obtain optimal images until observation of the rectus abdominis. We then moved the probe anterolaterally to obtain a transverse view of the abdominal layers, scanning the abdominal wall between the iliac crest and the subcostal margin. This allowed for visualization of the external oblique, internal oblique, transversus abdominis, and, most deeply, the peritoneal cavity.

This technique took approximately 5–10 min (each side). A systematic time recording was not performed.

### 2.4 Transabdominal preperitoneal inguinal hernia repair (TAPP)

More than 10 different surgeons conducted the TAPP IHR in this study. Next to an umbilical 10-mmport for the 30° telescope, two 5-mm ports, 5 cm from umbilicus at the lateral borders of the rectus abdominis muscle were inserted. CO_2_ to a pressure of 15 mmHg was insufflated. After hernial sac reduction, a polypropylene mesh (Medtronic®) was inserted through the 10-mm port. The mesh was placed and covered the entire myopectineal orifice. The mesh was not fixated. While lowering the intra-abdominal pressure to 8 mm Hg, the inguinal canal was closed by a running dissolvable suture.

### 2.5 Statistical analysis

Analysis was done using R (ver. 3.6.1). Data was presented as a number (percentages) for nominal, or as a mean ± SD/median (min-max) for metric variables. For the comparison of nominal variables between groups, Fishers exact test was used for metric variables. Normality was tested by using the Shapiro-Wilk test, and hence the T-test or the Wilcoxon-test. A p-value < 0.05 was considered as statistically significant. No corrections for multiple testing was done.

### 2.6 Aims

With the exception of clavien-dindo-classification (CDC [14]) and length of hospital stay, all endpoints were measured on the day of operation (DOO).

The primary endpoint was the analgesic cumulative need medication (CNM) of opioids. The secondary endpoints were the CNM of metamizole, NSAIDs, and acetaminophen, all relevant complications according to CDC during the hospital stay, operating time, and length of hospital stay. Another secondary endpoint was the pain level in the PACU using the VAS score (visual analog scale). Individuals were asked at least once about their pain level in the PACU. In some cases, the patients were asked several times. The mean value was calculated in these cases.

All patients received metamizole 8 g/d, or in case of an allergy, ibuprofen 2400 mg/d as a standard analgesic medication.

### 2.7 Database

In April 2020, an MS Excel data sheet was provided. This data was imported into R (ver. 3.6.1), and multiple plausibility checks were performed. In May 2020, inconsistencies of the data were resolved.

## 3 Results

A total of 116 patients were enrolled (UG-TAP-Block group, n = 58, IVG-TAP-Block group, n = 58).

### 3.1 Univariate analysis on baseline characteristics

The univariate analysis on matching criteria and BMI is shown in Table 1.

**Table 1:** Univariate analysis on baseline matching criteria and BMI.

Variable		UG-TAP group n = 58	VG-TAP group n = 58	*p*-value
Age	*years*	59.0 (13.8)	58.8 (14.1)	0.926

Male sex		93.10% (n = 54)	93.10% (n = 54)	1.000

ASA preoperative	*I*	42% (n = 24)	48.6% (n = 24)	1.000
	*II*	48% (n = 29)	40.4% (n = 29)	
	*III*	9.95% (n = 5)	10.7% (n = 5)	
	*IV + V*	0% (n = 0)	0% (n = 0)	

BMI	*kg/m2*	25.7 (3.34)	26.5 (4.05)	0.269

Hernia size^a^	*I*	13.78% (n = 8)	13.78% (n = 8)	1.000
	*II*	84.48% (n = 49)	84.48% (n = 49)	
	*III*	5.17% (n = 3)	5.17% (n = 3)	

ASA = American Society of Anesthesiologists physical status classification; BMI Body Mass Index.

Continuous measurements are presented as mean (SD); TAP transversus abdominis plane.

a According to EHS classification; UG ultrasound-guided; VG visual-guided.

### 3.2 Univariate analysis on perioperative data

Table 2 depicts the results of univariate analysis on perioperative data.

**Table 2:** Univariate analysis on perioperative data.

Variable		UG-TAP-Block group *n* = *58*	VG-TAP-Block group *n* = *58*	*p*-value
Hernia location				0.225
	*Left*	39.7% (n = 23)	34.5% (n = 20)	
	Right	43.1% (n = 25)	56.9% (n*=*33)	
	Bilateral	17.2% (n = 10)	8.6% (n = 5)	

Location of hernia orifice				0.924
	*Direct unilateral*	*7*	*8*	
	Indirect unilateral	39	35	
	Combined unilateral	4	7	

CDC Grading	None	100% (n = 58)	98.27% (n = 57)	>0.999
	NA	0% (n = 0)	1.72% (n = 1)	
	I–V	0% (n = 0)	0% (n = 0)	

Operating time	*minutes*	50.86 (17.6)	51.94 (SD 24.7)	0.786

LOS	*Days*	2 (0)	2.02 (0.13)	0.322

CDC Clavien-dindo classification; Continuous measurements are presented as mean (SD); TAP transversus abdominis plane; NA not applied.

### 3.3 Univariate analysis on primary and secondary endpoints

#### 3.3.1 Primary endpoint: CNM of opioids

Patients in the UG-TAP-Block received an average of 10.2 mg (SD 5.09) of morphine and oxycodone on the day of their operation. Individuals of IVG-TAP-Block group received an average of 8.71 mg (SD 5.34) of morphine and oxycodone on the day of their operation (p = 0.134).

Patients in the UG-TAP-Block received an average of 0.38 mg (SD 1.46) of piritramide on the day of their operation. Individuals in the IVG-TAP-Block group received an average of 0.71 mg (SD 2.73) of piritramide on the day of their operation (p = 0.423; Table 3).

**Table 3:** Univariate analysis on pain and cumulative need analgesic medication.

Variable		UG-TAP group *n* = *58*	VG-TAP group *n* = *58*	*p*-value
Pain level in the PACU	*VAS-Score*	0.67 (1.26)	0.84 (1.42)	0.492
CNM of acetaminophen	*G*	0.00 (0.00)	0.03 (0.26)	0.322
CNM of NSAR agents	*G*	11.6 (79.0)	6.21 (18.4)	0.618
CNM of metamizol	*G*	1.29 (0.96)	1.68 (0.70)	**0.015**
CNM of morphin and oxycodon	*mg*	10.2 (5.09)	8.71 (5.34)	0.134
CNM of piritramid	*mg*	0.38 (1.46)	0.71 (2.73)	0.423
No CNM required	*%*	0 (0)	0 (0)	1.000

CNM Cumulative need medication; Continuous measurements are presented as mean (SD); NSAR non-steroidal anti-rheumatic; PACU Post-anesthesia care unit; TAP Transversus abdominis plane; VAS Visual analog scale.

#### 3.3.2 Secondary endpoint: CNM of acetaminophen, metamizole and nonsteroidal anti-inflammatory drugs (NSAIDs)

Patients in the UG-TAP-Block group received no acetaminophen on the day of their operation. Individuals of IVG-TAP-Block group received an average of 0.03 g (SD 0.26) of acetaminophen on the day of their operation (p = 0.322).

Patients in the UG-TAP-Block group received an average of 1.29 g (SD 0.96) of metamizole on the day of their operation. Individuals of the IVG-TAP-Block group received an average of 1.68 g (0.70) of metamizole on the day of their operation (p = 0.015).

Patients in the UG-TAP-Block group received an average of 11.6 g (SD 79.0) of NSAIDs on the day of their operation. Individuals of the IVG-TAP-Block group received an average of 6.21 g (SD 18.4) of NSAIDs on the day of their operation (p = 0.618; Table 3).

#### 3.3.3 Secondary endpoint: pain after surgery in the PACU on the day of operation

Using a VAS score, patients of the UG-TAP-Block group stated an average pain level of 0.67 (SD 1.26) in the PACU. Individuals in the IVG-TAP-Block group stated an average pain level of 0.84 (SD 1.42) in the PACU (p = 0.492; Table 3).

#### 3.3.4 Secondary endpoint: postoperative complications

A total of 58 patients (100%) in the UG-TAP-Block group had no complications (CDC = 0). No patient suffered from a CDC I–V complication. No TAP-Block related major complication has been detected. (Table 2).

A total of 57 patients (98.27%) in the IVG-TAP-Block group had no complications (CDC = 0). In one case, the data was not applied. No patient suffered from a CDC I–V complication. No TAP-Block related major complication has been detected.

#### 3.3.5 Secondary endpoint: LOS

The average LOS was 2.00 days (SD 0) for the UG-TAP-Block group, and 2.02 days (SD 0.13) for IVG-TAP block group (p = 0.322; Table 2).

#### 3.3.6 Secondary endpoint: operating time

The average operating time was 50.86 (SD 17.6) for the UG-TAP-Block group, and 51.94 (SD 24.7) for the IVG-TAP block group (p = 0.786; Table 2).

## 4 Discussion

The TAP block prior to IHR in TAPP technique led to a substantial increase of early postoperative pain and a reduction of painkiller consumption. Moreover, a reduced consumption of opioid agents may prevent potential temporary intraoperative brain tissue oxygen desaturation, which influence mental state and pain intensity estimation during the post-op period when assessed by VAS. Stebelski et al. (2010) conducted a blinded randomized clinical trial among 30 individuals. The patients in the TAP-Block group (n = 15) suffered from less pain within 24 h after TAPP inguinal hernia repair [8]. Moreover, our study group retrospectively analyzed 838 individuals. The TAP-block group consisted of 364 individuals [3]. On the DOO, these patients needed significantly less painkillers and suffered from less pain after IHR in TAPP technique (control group n = 402). Additionally, the IVG-TAP-Block was compared to the UG-TAP-Block. No differences occurred in terms of CDC, pain level in the post-anesthesia care unit (PACU), and consumption of painkillers. However, the groups were not further analyzed in terms of biometric and perioperative data. To reveal more evidence, we performed the matched pair analysis at hand.

The chosen approach may play a role in prevention of TAP block related complications. To our knowledge, at least four TAP block related complications have been reported [[15], [16], [17], [18]]. In two cases (liver trauma and transient femoral nerve palsy), the TAP block with UG was performed [16,18]. Comparable complications were reported by Manatakis et al. (2013) and Farooq et al. (2008) [15,17]. In both cases, the TAP block was performed according to the landmark based “two-pop” technique (neither IVG- nor UG) described by McDonnell et al., in 2008 [19]. Our review of literature did not reveal any complications following IVG-TAP block [3]. Hence, it can be assumed that the IVG-TAP allows a safe infiltration, without unintentional organ injuries. The needle can be visualized during the laparoscopy. However, experienced surgeons can detect the muscle layers by ultrasound without risking any damage. In two cases, a transient femoral nerve palsy was described [17,18]. The authors assumed that a combination of high concentration of local anesthetic and the pressure of the abdominal binder (after caesarean delivery) resulted in the development of this complication. This injury naturally may occur following IUG-TAP block. Regardless of the chosen approach, the TAP block remains a safe procedure with only a few case reports with minor complications.

In general, it has been frequently elaborated that the TAP block may lead to a reduced analgesic use following IHR [3,20]. To our knowledge, the IVG and UG have not been compared further to one another in terms of analgesic use postoperatively. Among both groups the consumption of morphine, oxycodone, piritramide, NSAIDs and acetaminophen did not significantly differ from each other. Nevertheless, individuals in the UG-TAP group used significantly less metamizole (UG-TAP group 1.29 g (0.96); IVG-TAP-group 1.68 g (0.70), p = 0.015). Thus, patients in the UG group had a lower analgesic use. This may reflect the fact, that the UG-TAP facilitates a more precise anesthetic injection in the proper layer. Individuals in the UG group showed a decreased pain level, without statistical significance. VAS-Score: UG-TAP group 0.67, IVG-TAP group 0.84).

We aimed to further investigate the possible superiority of the UG-TAP. Hence, we reviewed the literature for the TAP block use prior to laparoscopic ventral hernia repair. Our search yielded three relevant publications including a matched pair analysis (n = 52) on our own [21]. Two prospective randomized clinical trials (n = 80, laparoscopic ventral hernia repair in IPOM technique) revealed a significant pain reduction effect within 24 h after surgery [22,23]. On the contrary to that, our study group and Fields et al. (2015) did not detect an IVG-TAP block impact on early postoperative pain and analgesic use [21,24]. The authors performed a randomized trial among 100 individuals who underwent laparoscopic ventral hernia repair in IPOM technique. These findings may confirm our assumption, that the UG-TAP block is more effective. But like mentioned prior to laparoscopic IHR (TAPP + TEP), a total of 6 studies (n = 1089) demonstrated an impact of the UG- and IVG-TAP block on pain reduction and analgesic use [3,[7], [8], [9], [10], [11]]. Further prospective clinical trials are mandatory to reveal more evidence.

A systematic time recording has not been carried out. Nevertheless, to our experience, the IVG-TAP block took us no more than 1 min (on each side). In comparison, at least 5 min were needed for the UG-TAP most likely due to covering and disinfecting the integumentary field. Additional time was needed for the ultrasound head covering. From an economic perspective, the time benefit of the IVG-TAP might be important. Also, in terms of material cost, the intraoperative approach appears to be superior in comparison to the UG-TAP (drapes, additional disinfectants, and sterile gloves).

Although a matching of the individuals was performed, the retrospective study design with a lack of a long-term follow-up and the sample size naturally remains a study limitation. A systematic time and cost recording of the TAP block have not been carried out. Our assumptions on that topic have been solely on our experiences. More than 10 different surgeons conducted the TAPP IHR in this study. It increases the bias of the results as well.

## 5 Conclusions

Due to our findings, we assume that the UG-TAP-Block may reduce postoperative pain and analgesic consumption more effectively than the VG-TAP-Block. The ultrasound may facilitate a more precise injection of the local anesthetic in the right layer. On the contrary, the IVG-TAP block appeared to be less time and cost consuming.

Further prospective clinical trials are necessary to confirm our assumption.

## Disclosure statement

CP, NB, JF, GD, NA, PO, UG, KB, SA, HF and MS declare no conflict of interest.

## Funding

No funding has been received.

## Provenance and peer review

Not commissioned, externally peer reviewed.

## Declaration of competing interest

None.

## Taril registrationnumber

The study was registered with the German clinical trial registry DRKS (DRKS00021679). No funding has been received.

https://www.drks.de/drks_web/navigate.do?navigationId=search&reset=true.
